# Targeted Motor Control Considering Sternal Position Improves Spinal Alignment in Pregnant Women at Risk for Preterm Birth with Low Back Pain

**DOI:** 10.3390/jcm13247661

**Published:** 2024-12-16

**Authors:** Arkadiusz Łukasz Żurawski, Sun Young Ha, Grzegorz Świercz, Olga Adamczyk Gruszka, Wojciech Piotr Kiebzak

**Affiliations:** 1 Collegium Medicum, Jan Kochanowski University in Kielce, al. IX Wieków Kielc 19a, 25-516 Kielce, Poland; wojciech.kiebzak@ujk.edu.pl; 2 Health & Wellness Research Institute, Kyungnam University, 7 Kyungnamdaehak-ro, Masanhappo-gu, Changwon-si 51767, Gyeongsangnam-do, Republic of Korea; mallows205@naver.com; 3 Clinic of Gynecology and Obstetrics, Provincial Complex Hospital, Grunwaldzka 45, 25-736 Kielce, Poland; grzegorz.swiercz60@gmail.com (G.Ś.); oadamczyk@ujk.edu.pl (O.A.G.)

**Keywords:** low back pain, pregnancy, position of the sternum, spinal alignment, posture

## Abstract

**Background:** Lumbopelvic pain (LBP) is a prevalent condition during pregnancy, affecting a significant proportion of pregnant women. It arises from hormonal, biomechanical, and postural changes, often exacerbating discomfort and impairing quality of life. This study aimed to evaluate the effects of targeted motor control interventions focusing on sternal alignment on spinal alignment, pain, and muscle activity in pregnant women at risk of preterm birth. **Methods:** This pre–post quasi-experimental study included 32 pregnant women at 28–32 weeks of gestation, who were hospitalized due to the risk of preterm birth. Inclusion criteria required participants to have LBP lasting at least two weeks and the ability to walk and stand for 40 min. The intervention involved targeted motor control exercises designed to optimize sternal and sacral alignment. Spinal alignment, pain intensity, and muscle activity were measured pre- and post-intervention using the DIERS formetric system, numerical rating scale (NRS), and electromyography (EMG), respectively. Data were analyzed using Wilcoxon signed-rank tests. **Results:** Significant improvements were observed in spinal alignment parameters, including reductions in the sternal angle, sacral angle, cervical and lumbar lordosis depths, thoracic kyphosis angle, and pelvic tilt (*p* < 0.05). Pain intensity decreased significantly from a mean NRS score of 5.77 ± 1.42 in the relaxed posture to 2.54 ± 0.71 in the corrected posture (*p* < 0.05). Muscle activity of the rhomboid muscles increased in the corrected posture, correlating with improved thoracic kyphosis, while activity of the serratus anterior muscle showed reductions (*p* < 0.05). **Conclusions:** Targeted motor control focusing on sternal alignment effectively improved spinal alignment and reduced pain in pregnant women at risk of preterm birth with LBP. The intervention offers a safe, non-invasive, and practical approach to managing pregnancy-related musculoskeletal challenges. Future research should validate these findings in diverse populations and explore long-term effects and broader clinical applications.

## 1. Introduction

Lumbopelvic pain (LBP) during pregnancy is a prevalent condition, affecting ap-proximately 50–70% of pregnant women [1,2]. This term encompasses discomfort in the lumbar spine, sacroiliac region, and pelvis, often resulting from hormonal, biomechanical, and postural changes specific to pregnancy [3,4]. Hormonal factors, particularly the increased secretion of relaxin, lead to joint laxity, which compromises pelvic stability and predisposes pregnant women to LBP [5]. Additionally, biomechanical changes, such as an anterior shift in the center of gravity, increased lumbar lordosis, and pelvic anteversion, contribute to mechanical loading on the lumbar spine and pelvic structures [6,7].

Postural alterations most frequently described during pregnancy include increased lumbar lordosis, thoracic kyphosis, and cervical lordosis, as well as pelvic anteversion and shoulder protraction [8,9]. These changes accommodate the growing fetus but can impair load transfer, strain pelvic ligaments, and exacerbate pain [10]. Moreover, LBP not only affects physical mobility but also significantly disrupts psychological well-being, daily activities, and quality of life [11]. Pregnant women with persistent LBP report limitations in performing routine tasks, reduced sleep quality, and heightened emotional distress [12].

### 1.1. Etiology and Clinical Significance

Addressing LBP during pregnancy is crucial, as effective management can improve maternal comfort and mitigate potential complications arising from physical and psychological stress [13]. Various interventions have been developed to manage pregnancy-related LBP, including exercise therapy, manual techniques, and postural correction strategies [14]. European guidelines recommend individualized exercise programs focusing on stabilization, posture control, and safe pain relief methods while avoiding pharmacological treatments unless absolutely necessary [15]. These conservative approaches, as highlighted by Gutke et al. as well as Liddle and Pennick, are considered safe and effective [16,17].

### 1.2. Spine Alignment and Its Role in LBP

Spinal alignment, as defined in this study, refers to the relative positioning of key spinal segments (cervical, thoracic, and lumbar) in relation to the pelvis. Proper alignment minimizes mechanical strain on the musculoskeletal system and optimizes load transfer during daily activities [18]. During pregnancy, significant changes in spinal alignment, such as increased lumbar lordosis and thoracic kyphosis, are recognized contributors to LBP [19,20]. This study evaluates these changes comprehensively, focusing on the interplay between the sternum and sacrum as markers of spinal and pelvic alignment. Targeted interventions aimed at restoring this alignment may reduce pain and enhance functional stability.

**Study objectives**

This study focuses on pregnant women at risk for preterm birth, a population chosen due to their frequent hospitalization and uniform clinical risk profile. However, the findings have broader implications for the general population of pregnant women experiencing LBP. The primary aim of this study is to evaluate the effectiveness of a targeted motor control intervention focused on sternal and sacral positioning in improving spinal alignment, reducing pain, and enhancing muscle activity in pregnant women with LBP. By addressing the unique biomechanical and postural challenges of pregnancy, this research contributes to the development of accessible, non-invasive strategies to improve maternal health and quality of life.

## 2. Materials and Methods

### 2.1. Participants

The study included 32 pregnant women receiving outpatient care at the Provincial Complex Hospital in Kielce, Poland. These participants were identified during routine medical visits or hospitalization in the Department of Pregnancy Pathology at the Clinic of Gynecology and Obstetrics. The inclusion of participants was based on clinical evaluation by attending obstetricians, who assessed their eligibility for the study using standard diagnostic protocols for high-risk pregnancies. Women considered at risk for premature birth were identified based on cervical shortening, episodes of uterine contractions, and additional clinical markers such as abnormal fetal fibronectin levels or a history of prior preterm birth. Participants were aged between 22 and 30 years, representing a population with relatively homogenous demographic and clinical profiles.

### 2.2. Inclusion and Exclusion Criteria

To ensure uniformity in the study population, the following criteria were applied:

- Inclusion Criteria:
  1. Pregnant women at 28–32 weeks of gestation diagnosed as being at risk of premature birth based on clinical markers.
  2. Women reporting low back pain (LBP) persisting for at least two weeks before study enrollment.
  3. Ability to walk and maintain a standing position for at least 40 min, which was deemed essential for the intervention and measurement protocols.
- Exclusion Criteria:
  1. Pregnant women diagnosed with gestational diabetes, as this condition could independently affect posture and physical activity.
  2. Pre-existing musculoskeletal abnormalities, such as scoliosis or joint deformities, which could bias outcomes.
  3. History of neuromuscular trauma or disease, which could interfere with motor control and muscle activity assessments.
  4. Diagnosed cardiovascular problems that might pose safety concerns during prolonged standing.

These criteria were verified through clinical records, direct interviews, and physical examinations conducted by the medical team. Table 1 summarizes the inclusion and exclusion criteria for ease of reference.

### 2.3. Health Monitoring and Ethical Considerations

The health statuses of both the participants and their fetuses were continuously monitored throughout the study by a team comprising two obstetricians and a midwife. Standardized protocols, including regular ultrasound examinations and cardiotocography, were used to ensure fetal well-being and participant safety. During the experiment, no adverse events or complications were reported for any participants or fetuses.

Participants were thoroughly informed about the study’s purpose, procedures, and potential risks. Written informed consent was obtained from all participants before their enrollment in the study. This study was conducted in accordance with the ethical principles outlined in the Declaration of Helsinki and approved by the Bioethics Committee of Jan Kochanowski University (approval number: 27/2022).

### 2.4. Study Design and Sample Size Calculation

This was a pre–post quasi-experimental study without a control group. The study design was chosen to prioritize participant safety by avoiding the ethical concerns associated with withholding intervention in a control group. The sample size was calculated using G*Power software (version 3.1.9.7, Heinrich-Heine-Universität Düsseldorf, Germany). A medium effect size (Cohen’s d = 0.5) was assumed based on prior studies investigating the effects of posture correction interventions for low back pain in pregnant women. With a statistical power of 0.8 and a significance level (α) of 0.05, the required sample size was determined to be 28 participants. To account for a potential dropout rate of 10%, the sample size was increased to 32 participants.

By addressing both clinical and logistical considerations, this study design and sample size calculation ensured adequate statistical power while maintaining participant safety and ethical standards.

### 2.5. Intervention Description

The intervention aimed to correct posture through targeted motor control, focusing on muscle activation and local restriction removal methods to improve spinal alignment and reduce low back pain (LBP). This intervention was based on a protocol adapted from Kiebzak et al. [21], adjusted specifically for the needs of pregnant women.

#### 2.5.1. Procedure for Posture Correction

Participants were guided through a step-by-step process to achieve a corrected posture, which involved the following.

Sternal Positioning: Participants were instructed to lift their sternum diagonally to approximately 64°, as measured relative to the horizontal plane. This angle was monitored visually by the examiner to ensure uniformity across all sessions. Participants maintained a neutral head position by aligning the mandible parallel to the floor.

Scapular Adjustment: Participants were taught to retract and depress their scapulae into a “back-and-down” position to counteract the protraction often caused by increased thoracic kyphosis during pregnancy.

Pelvic Alignment: The corrected sternal position naturally reduced anterior pelvic tilt, promoting physiological lumbar extension.

The examiner used visual inspection and palpation to verify the corrected posture. All instructions were delivered consistently by the same trained examiner, ensuring uniform application of the intervention.

#### 2.5.2. Validation of Muscle Activation

The effectiveness of muscle activation during posture correction was confirmed through palpation of key muscles, including the internal oblique, multifidus, rhomboid, serratus anterior, and external oblique. This qualitative assessment was supported by standardized EMG data collection, ensuring quantitative validation without introducing new procedures or literature references.

#### 2.5.3. Duration and Rationale

Each participant maintained both relaxed and corrected postures for 30 min, as this duration was deemed sufficient to induce observable biomechanical and physiological changes. While the 30-min duration exceeds the time most pregnant women report as comfortable, the participants were allowed brief adjustments or rest periods if discomfort arose, ensuring safety and compliance.

#### 2.5.4. Instruction and Education

Participants underwent a preliminary instructional session that included the following.

Demonstration: The examiner demonstrated the corrected posture, highlighting sternal positioning, scapular adjustment, and pelvic alignment.

Practice: Participants practiced under supervision until they could independently replicate the corrected posture. Emphasis was placed on clear verbal cues and consistent feedback to ensure comprehension.

#### 2.5.5. Reliability Measures

The following methods were employed to ensure consistency.

Standardized Approach: All posture corrections were performed and verified using a single protocol, with no variations introduced.

Examiner Training: The examiner underwent specialized training to ensure reliability in identifying anatomical landmarks and guiding participants.

### 2.6. Sternal and Sacral Angles

The sternal and sacral angles were measured using a Saunders digital inclinometer (Baseline Digital Inclinometer, The Saunders Group Inc., Chaska, MN, USA). To ensure consistency and reliability, the following standardized procedures were implemented.

Measurement Procedures

**Figure 1:**
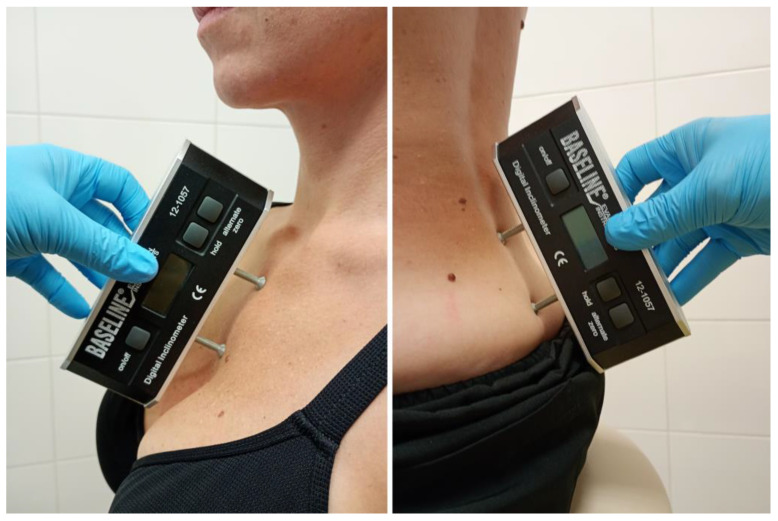
Measurement of the angle of the sternum and sacrum using a Saunders inclinometer.

Sternal Angle:

The inclinometer was placed on the front of the sternum body. The examiner ensured that the device’s foot was securely positioned along the midline of the sternum to avoid deviations. Participants were instructed to maintain a neutral standing position with their head aligned and gaze fixed forward [21,22] (Figure 1).

Sacral Angle:

The sacral angle was measured by placing one foot of the inclinometer on the sacroiliac region (posterior superior iliac spine) and the other foot along the medial sacral crest. To reduce variability, landmarks were palpated and marked prior to the measurement, ensuring accurate and consistent device placement across all participants [21,22] (Figure 1).

Standardization of Participant Stance

To minimize measurement errors, the following standards were employed.

Predefined Mat Setup: Participants stood on a 1 × 1 m predefined mat. This mat featured gridlines to guide foot placement and ensure parallel alignment of the feet at shoulder-width apart.

Fixed Reference Point: Participants faced a fixed visual reference point to maintain consistent orientation.

Postural Instructions: Participants were instructed to remain relaxed, with their weight evenly distributed between both feet.

Examiner Reliability

To ensure consistent measurement practices, the following standards were employed.

Training: The examiner underwent rigorous training to standardize the identification of anatomical landmarks.

Consistent Examiner: All measurements were conducted by the same examiner to eliminate inter-examiner variability.

Reliability and Validity Considerations

Previous studies have demonstrated the reliability of the Saunders digital inclinometer for measuring spinal and pelvic angles [21,22]. To further ensure validity, the equipment was calibrated weekly following the manufacturer’s guidelines. This practice minimized potential technical discrepancies and ensured accurate readings throughout the study.

Adjustments for Anatomical Variability

Anatomical differences among participants were accounted for by thoroughly palpating landmarks before placing the inclinometer. This ensured accurate placement regardless of individual variability in body composition or postural alignment.

### 2.7. Spinal Angle

The DIERS formetric system (DIERS Formetric 4D, DIERS International GmbH, Schlangenbad, Germany) was utilized for precise and non-invasive measurement of spinal angles. This advanced system enables digital 3D reconstruction and scanning of the spine without the use of radiation or markers, ensuring participant safety and measurement accuracy [23].

Measurement Protocol

Participant Positioning:

Participants stood barefoot on a designated platform, 2 m away from the DIERS formetric system. The following instructions were provided to standardize posture.

Feet were aligned shoulder-width apart, parallel, and positioned symmetrically to the scanner’s field of view.

Participants were asked to relax their arms and maintain a neutral gaze toward a fixed point to minimize upper body movement.

Scanning Procedure:

Relaxed Posture: The initial scan captured the participant’s natural posture without corrections.

Corrected Posture: The second scan recorded the participant’s posture after applying targeted motor control interventions.

Each scanning session lasted 6 s, during which participants were instructed to remain still and breathe naturally to avoid postural fluctuations.

Parameters Measured

The following spinal parameters were automatically computed using the DIERS formetric software.

Kyphosis Angle: The curvature of the thoracic spine.

Lordosis Angle: The curvature of the lumbar spine.

Depth of Cervical Lordosis: The horizontal distance between the peak of cervical curvature and the thoracic plumb line.

Depth of Lumbar Lordosis: The horizontal distance between the peak of lumbar curvature and the thoracic plumb line.

Pelvic Tilt Angle: The inclination of the pelvis relative to the horizontal plane.

Reliability and Calibration

To ensure the reliability and accuracy of the measurements, the following standards were applied.

Examiner Training: The same examiner conducted all scans to eliminate inter-examiner variability. The researcher has a certificate confirming his skills in clinical assessment using the DIERS system.

Calibration: The DIERS formetric system underwent weekly calibration as per the manufacturer’s guidelines, ensuring consistent performance.

Validation of the Device

The DIERS formetric system has been validated in clinical and research settings as a reliable tool for measuring spinal parameters. The system’s high-resolution imaging and automated software minimize human error and provide accurate reconstructions of spinal curvature and alignment [23].

Considerations for Anatomical and Positional Variability

To account for potential variability, the following standards were applied.

Landmark Identification: The DIERS system automatically identifies key anatomical landmarks (e.g., vertebral prominences, sacral midpoint), ensuring consistent measurements across participants.

Postural Adjustments: Participants were reminded to relax during the relaxed posture scan and actively engage during the corrected posture scan to reflect the intended spinal alignment changes.

**Figure 2:**
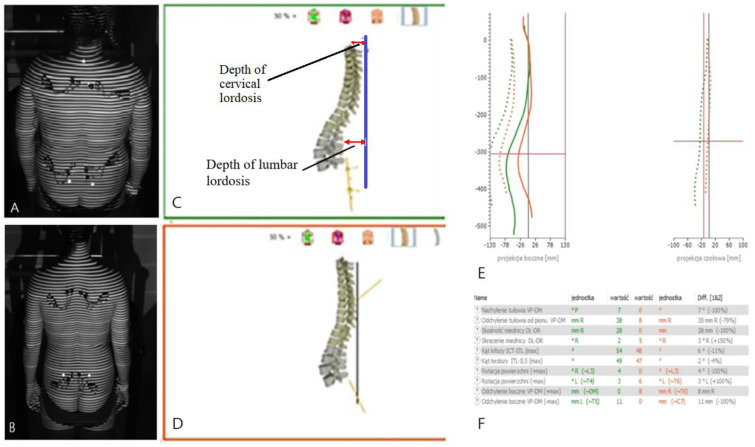
Measurement of spine parameters using the DIERS formetric system. (**A**)—patient in a relaxed position, (**B**)—patient in a corrected position, (**C**)—image of the spine in a relaxed position, (**D**)—image of the spine in a corrected position, (**E**)—comparison of the shape of the spine in a relaxed and corrected position, (**F**)—comparison of parameters describing the shape of the spine in a relaxed and corrected position.

An accurate representation of the results is shown in Figure 2.

### 2.8. Pain

Pain intensity was assessed using the numerical rating scale (NRS), a widely recognized and validated tool for evaluating subjective pain levels [24]. On this scale, 0 represents “no pain at all”, and 10 signifies “the greatest pain imaginable”. This approach ensured a simple yet effective means of capturing participants’ pain experiences.

Measurement Protocol

Timing of Pain Assessments:

Pain levels were measured at two distinct time points for each participant:

After maintaining a relaxed posture for 30 min.

After maintaining a corrected posture following the targeted motor control intervention, also for 30 min.

These measurements allowed for a direct comparison of pain intensity between the two postural conditions.

Standardization of Measurements:

To minimize variability, the following standards were applied.

Participants were instructed to report their pain levels immediately after each posture session.

The same examiner administered the NRS to all participants, ensuring consistency in instructions and recording.

Consideration for Fluctuations in Pain Perception:

Participants were given clear guidance to focus on their overall pain experience during each session, avoiding momentary fluctuations caused by distractions or movement.

Rationale for the 30-min Duration

The 30-min duration for maintaining each posture was chosen based on clinical observations indicating that pregnant women with low back pain frequently report discomfort after 10–15 min of standing. Doubling this time ensured the following:

Pain was induced to a measurable degree, enabling meaningful comparisons.

Participant safety and comfort were prioritized, with frequent monitoring during the sessions.

Reliability of the NRS

The NRS has been extensively validated for use in both clinical and research settings. Its simplicity, ease of administration, and ability to provide reliable and reproducible data make it particularly suited for populations experiencing acute or chronic pain, such as pregnant women with low back pain [24].

Ethical Considerations

Given the subjective nature of pain, participants were informed of the following:

They could withdraw from the posture session at any time if pain became intolerable.

Reporting accurate pain levels was crucial for the study’s integrity and their own comfort management.

Limitations

While the NRS provides valuable insights into pain intensity, it does not capture other dimensions of pain, such as emotional or psychological impact. Future studies could complement the NRS with multidimensional pain assessment tools to provide a more comprehensive understanding.

### 2.9. Muscle Activity

To assess muscle activity, a 16-channel electromyography (EMG) system (Noraxon, DTS, Desktop Direct Transmission System, Scottsdale, AZ, USA) was used. This advanced system ensured high precision in capturing and analyzing the electrical activity of targeted muscles during relaxed and corrected postures.

Measurement Protocol

Signal Acquisition and Processing:

The EMG signals were recorded at a sampling rate of 1000 Hz, with a band-pass filter set to 20–500 Hz to exclude noise and irrelevant frequencies.

Signals were processed using MyoResearch XP Master Edition 3.18.136 software (Noraxon Inc., Scottsdale, AZ, USA). Raw data underwent full wave rectification and were smoothed using a root mean square (RMS) algorithm with a window of 100 ms to standardize variability.

Normalization to Referenced Voluntary Contraction (RVC):

Muscle activity was normalized to referenced voluntary contraction (RVC) for comparability across participants.

RVC was determined during a quiet standing posture maintained for 5 s. During this period, participants were instructed to avoid unnecessary movements, ensuring stable baseline measurements [21].

Activity during the corrected posture was recorded for 5 s, and the first and last 1 s of data were excluded to eliminate potential artifacts during the transition to and from the posture. The middle 3 s of data were used for analysis [21].

Measured Muscles:

The following muscles were assessed bilaterally:

Internal oblique

Multifidus

Rhomboid

Serratus anterior

External oblique

These muscles were selected based on their known roles in spinal stability, posture maintenance, and their potential involvement in pregnancy-related postural adaptations.

Calibration and Consistency:

All measurements were conducted by the same trained examiner to minimize inter-rater variability.

The EMG equipment was calibrated regularly according to the manufacturer’s guidelines to ensure accuracy.

Skin preparation included cleaning the skin with alcohol and shaving, if necessary, to reduce impedance and enhance signal quality.

Rationale for Muscle Selection

The chosen muscles are integral to maintaining spinal alignment and pelvic stability:

The multifidus and internal oblique are key stabilizers of the lumbar spine.

The rhomboid supports thoracic kyphosis and scapular positioning.

The serratus anterior and external oblique are involved in scapular movement and trunk rotation, respectively. These muscles are often affected by the biomechanical and postural changes of pregnancy, which can contribute to low back pain.

Reliability and Validation

The reliability of the EMG system was ensured using the following standards.

Regular calibration before and during the study.

A standardized protocol for sensor placement based on international recommendations (e.g., SENIAM guidelines).

Verification of electrode placement via palpation during muscle activation tasks.

Ethical Considerations

Participants were informed about the EMG procedures and assured of their safety. Written consent was obtained before applying sensors. All measurements were conducted in a comfortable, climate-controlled setting to reduce external influences on muscle activity.

Limitations

While the %RVC normalization method accounts for individual variability, differences in fatigue or discomfort between relaxed and corrected postures could still influence the measurements. Future studies could explore additional normalization methods or longer-duration recordings to provide a more comprehensive view of muscle activity.

All measurements were conducted by the same trained and experienced examiner to ensure consistency and minimize inter-operator variability. The examiner underwent a standardized training program to ensure proper use of the equipment and adherence to measurement protocols. The reliability of the Saunders digital inclinometer and DIERS formetric system has been extensively validated in previous studies, demonstrating high inter-rater and intra-rater reliability for spinal alignment measurements under controlled conditions [22]. To further confirm consistency in this study, a pilot test was conducted on a separate group of participants before the main study to establish procedural accuracy and refine the measurement techniques.

Regular calibration of the equipment was performed prior to each measurement session according to the manufacturer’s guidelines. For the inclinometer, this included verifying baseline zeroing and confirming mechanical stability. For the DIERS formetric system, calibration involved ensuring proper alignment of the optical components and software validation checks to detect any system irregularities. These procedures ensured the precision and repeatability of all measurements.

Additionally, to ensure standardized participant positioning during measurements, the following methods were employed.

A predefined measurement area with consistent lighting and floor markings was used to avoid variability caused by environmental factors.

Participants were instructed to maintain a relaxed standing posture with feet aligned to specific markers to minimize deviations during successive scans.

Any deviations during the measurement process, such as participant movement or equipment anomalies, were documented, and the measurement was repeated to maintain data integrity.

## 3. Results

### 3.1. Alignment

The analysis revealed significant differences in spinal alignment parameters between the relaxed and corrected postures. The angles of the sternum, sacrum, cervical lordosis, lumbar lordosis, kyphosis, and pelvic tilt were consistently larger in the relaxed posture than in the corrected posture (*p* < 0.05) (Table 2). These findings suggest that the corrected posture effectively reduces the excessive spinal curvatures and pelvic tilt typically observed in pregnant women with LBP.

Interpretation of Results:

Sternal and Sacral Angles: The reduction in the sternal angle (mean difference: 5.00 ± 1.43°) and sacral angle (mean difference: 7.19 ± 1.45°) in the corrected posture indicates improved spinal alignment, reducing anterior pelvic tilt and enhancing load distribution along the spine.

Cervical and Lumbar Lordosis Depths: The significant decrease in cervical lordosis depth (mean difference: 20.45 ± 3.26 mm) and lumbar lordosis depth (mean difference: 11.35 ± 3.12 mm) reflects a reduction in the exaggerated curvatures often associated with LBP during pregnancy.

Kyphosis and Lordosis Angles: The decrease in the kyphosis angle (mean difference: 6.12 ± 1.31°) and lordosis angle (mean difference: 6.64 ± 1.17°) highlights the role of posture correction in mitigating thoracic and lumbar compensatory curves.

Pelvic Tilt: The reduction in pelvic tilt (mean difference: 4.83 ± 1.28°) suggests improved pelvic alignment, reducing stress on the lumbosacral region.

Clinical Relevance:

These results align with the hypothesis that targeted motor control interventions focusing on sternal and sacral positioning can optimize spinal alignment and potentially reduce mechanical stressors contributing to LBP. The observed changes in spinal and pelvic parameters demonstrate the biomechanical impact of posture correction, supporting its inclusion in therapeutic strategies for pregnant women with LBP.

Statistical Considerations:

To ensure robustness, statistical analyses included Wilcoxon signed-rank tests due to non-normal data distribution confirmed by the Kolmogorov–Smirnov test. The consistent *p*-values (<0.05) across all variables underscore the effectiveness of the intervention.

### 3.2. Pain

The analysis revealed a significant reduction in pain intensity between the relaxed and corrected postures, as measured using the numerical rating scale (NRS). The mean pain score decreased from 5.77 ± 1.42 in the relaxed posture to 2.54 ± 0.71 in the corrected posture (*p* < 0.05). These findings indicate that adopting a corrected posture can substantially alleviate pain perception in pregnant women with LBP (Table 3).

Interpretation of Results:

The observed reduction in pain intensity suggests that the corrected posture relieves biomechanical stress on the lumbar and pelvic regions, which are commonly implicated in pregnancy-related LBP.

The significant difference in NRS scores underscores the potential of targeted motor control interventions to provide immediate pain relief through improved spinal alignment.

Clinical Relevance:

Pregnant women frequently experience heightened pain due to postural imbalances and increased mechanical stress during daily activities. Correcting posture through targeted interventions may not only reduce pain but also improve functional comfort during pregnancy, allowing better engagement in routine tasks.

Statistical Considerations:

The Wilcoxon signed-rank test was used to compare NRS scores between postures, as the data distribution did not meet normality assumptions. The statistically significant *p*-value (<0.05) validates the efficacy of the intervention in reducing perceived pain.

Limitations and Future Considerations:

The NRS is a subjective measure of pain. While it provides valuable insight into participant-reported outcomes, future studies may benefit from incorporating objective measures, such as pressure-pain thresholds, to corroborate these findings.

The study did not account for individual variability in pain tolerance or the potential influence of external factors, such as physical activity or stress, which may affect pain perception.

### 3.3. Muscle Activity

The analysis of muscle activity revealed significant differences between relaxed and corrected postures for specific muscle groups. The activity of the right and left rhomboid muscles was significantly greater in the corrected posture compared to the relaxed posture (*p* < 0.05). Conversely, the activity of the left serratus anterior was significantly higher in the relaxed posture than in the corrected posture (*p* < 0.05) (Table 4).

Interpretation of Results:

- Rhomboid Muscles: The increased activity of the rhomboid muscles in the corrected posture suggests that these muscles play a crucial role in maintaining thoracic alignment. Their activation may counteract the protraction of the shoulders observed in the relaxed posture, thus improving overall spinal alignment.
- Serratus Anterior Muscle: The reduced activity of the left serratus anterior in the corrected posture could indicate a decrease in compensatory stabilization required in the relaxed posture. This finding aligns with the hypothesis that postural correction redistributes the mechanical load more efficiently across the musculoskeletal system.

Statistical Considerations:

The Wilcoxon signed-rank test was utilized for paired comparisons, as the muscle activity data did not follow a normal distribution. Significant *p*-values (<0.05) confirm the observed changes in muscle activation patterns for the rhomboid and serratus anterior muscles.

Clinical Implications:

- The increased activation of the rhomboid muscles during corrected posture highlights their importance in improving postural stability. This activation may also contribute to pain relief by reducing mechanical strain on the thoracic and lumbar regions.
- The observed reduction in serratus anterior activity in the corrected posture may reflect a shift in load distribution that reduces reliance on this muscle for compensatory stabilization.

Limitations and Future Directions:

- The normalization of EMG signals to %RVC ensures comparability across participants; however, individual variations in muscle strength and recruitment patterns may influence the results.
- Future studies could investigate the bilateral asymmetry observed in muscle activation patterns, particularly the differences between the right and left sides of the body.

## 4. Discussion

Safe and effective interventions are crucial for improving pain and posture in pregnant women at risk of preterm birth with LBP. This study demonstrated that targeted motor control focusing on sternal alignment led to significant improvements in spinal alignment, pain reduction, and muscle activity, offering a promising non-invasive approach for managing LBP in this vulnerable population.

### 4.1. Spinal Alignment

The mutual relationship between the sternum and sacrum plays a critical role in maintaining proper spinal alignment. In this study, relaxed posture exhibited larger sternal and sacral angles, as well as increased cervical lordosis, lumbar lordosis, and thoracic kyphosis. These findings are consistent with the existing literature, which highlights that pregnancy-related changes in spinal curvature contribute to postural instability and musculoskeletal discomfort [2,3]. For example, Bullock et al. reported a 6° increase in thoracic kyphosis during pregnancy, corroborating the observed trends in this study [13]. Corrected posture, achieved through targeted motor control, resulted in a decrease in these angles, with the sternal angle approximating the optimal alignment of 64° suggested by Kiebzak et al. [17].

The observed reduction in lumbar lordosis and thoracic kyphosis through sternal angle correction underscores the biomechanical impact of this intervention. By reducing anterior pelvic tilt and spinal overextension, both commonly observed during pregnancy, targeted motor control may alleviate mechanical stress on the lumbar spine and pelvic structures [20]. These findings suggest that posture correction can effectively mitigate postural instability and associated discomfort, contributing to better spinal health in pregnant women [1,19].

### 4.2. Pain

This study confirmed a significant reduction in pain levels following postural correction. Increased lumbar lordosis, often observed during pregnancy, heightens tension in the lumbosacral region and exacerbates pain [3,19]. By improving spinal alignment through targeted motor control, this study demonstrated a safe and effective non-pharmacological approach to pain relief. This finding is particularly relevant for pregnant women at risk of preterm birth, where medication options may be restricted due to potential risks to the fetus [20].

The alleviation of pain through postural correction may also reflect the redistribution of mechanical loads and a reduction in soft tissue strain. These results highlight the clinical relevance of targeting spinal alignment as part of a broader strategy to improve maternal well-being during pregnancy [14,17].

### 4.3. Muscle Activity

The results indicated increased activation of the rhomboid and serratus anterior muscles in the corrected posture. The increased activity of the rhomboid muscle, associated with thoracic kyphosis correction, suggests improved postural stability. This aligns with previous findings that emphasize the importance of thoracic muscle engagement in maintaining an upright posture [23]. However, the lack of significant changes in the multifidus and abdominal muscles warrants further investigation, as these muscles are also implicated in core stability and spinal health [22,24].

This study also highlights the role of upper-body alignment in overall spinal mechanics. The findings support the importance of integrating visual, vestibular, and somatosensory inputs to maintain a physiological spinal silhouette [25,26]. Future research could explore the impact of a more comprehensive intervention that includes targeted exercises for the lower back and abdominal regions.

### 4.4. Study Limitations

Despite the promising results, this study has several limitations:

1. Lack of a control group: Without a control group, it is challenging to attribute the observed effects solely to the intervention.
2. Short intervention duration: The study focused on immediate effects, and long-term benefits were not evaluated.
3. Homogeneous sample: Participants were exclusively pregnant women at risk of preterm birth, limiting the generalizability of the findings to other populations.
4. Uncontrolled factors: Variables such as occupation, pre-pregnancy physical activity, and weight gain during pregnancy were not accounted for, which may have influenced the results.

### 4.5. Future Directions

To address these limitations, future studies should focus on the following:

- Include a control group to strengthen causal inferences.
- Explore the long-term effects of targeted motor control on pain and posture.
- Enroll a more diverse participant pool to enhance generalizability.
- Investigate additional factors such as occupation, physical activity levels, and weight gain during pregnancy.

By addressing these gaps, future research can further validate and refine the application of targeted motor control for managing LBP in pregnant women, ultimately contributing to improved maternal health and quality of life.

## 5. Conclusions

This study demonstrates that targeted motor control interventions focusing on sternal alignment can effectively improve spinal alignment and reduce pain in pregnant women at risk of preterm birth with LBP. The intervention is non-invasive, safe, and practical, addressing specific biomechanical challenges associated with pregnancy without relying on pharmacological treatments. These findings are particularly relevant for pregnant women where limited medical options are available due to the risks associated with preterm birth.

However, due to the short duration of the intervention and the lack of a control group, the results should be interpreted with caution. The observed improvements reflect short-term associations rather than causal relationships, emphasizing the need for further research. Future studies should incorporate control groups, evaluate the long-term effects of this intervention, and examine its applicability to diverse populations, including those with varying levels of physical activity, pre-existing musculoskeletal conditions, and different pregnancy-related complications.

Expanding the scope of research to include additional musculoskeletal outcomes and the role of upper-body alignment in overall posture could provide a more comprehensive understanding of the intervention’s benefits. Such efforts would contribute to refining strategies for managing pregnancy-related LBP and improving maternal health outcomes more broadly.

## Figures and Tables

**Table 1:** Inclusion and exclusion criteria.

Criterion	Inclusion	Exclusion
Gestational Age	28–32 weeks	Outside this range
Low Back Pain	Lasting at least 2 weeks	None
Physical Ability	Able to stand and walk as required	Impaired mobility
Health Conditions	No contraindications	Gestational diabetes, musculoskeletal abnormalities, neuromuscular trauma, or cardiovascular problems

**Table 2:** Comparison of spinal, sternal, and sacral angles.

Variables	Relaxed Posture	Corrected Posture	Z	*p*
Sternal angle (°)	72.65 ± 6.17	67.65 ± 4.74	3.822	0.000 *
Sacral angle (°)	77.61 ± 7.58	70.42 ± 6.13	4.285	0.000 *
Depth of cervical lordosis (mm)	61.87 ± 17.45	41.42 ± 17.62	4.406	0.000 *
Depth of lumbar lordosis (mm)	56.34 ± 18.16	44.99 ± 16.22	4.000	0.000 *
Kyphosis angle (°)	56.38 ± 9.50	50.26 ± 10.26	4.406	0.000 *
Lordosis angle (°)	50.32 ± 9.75	43.68 ± 10.62	4.127	0.000 *
Pelvic tilt (°)	25.90 ± 6.77	21.07 ± 8.32	4.330	0.000 *

Results marked with * are statistically significant for *p* < 0.05.

**Table 3:** Comparison of the numerical rating scale within the group.

Variables	Relaxed Posture	Corrected Posture	Z	*p*
Pain	5.77 ± 1.42	2.54 ± 0.71	4.457	0.000 *

Results marked with * are statistically significant for *p* < 0.05.

**Table 4:** Comparison of muscle activity within the group (%).

Variables	Relaxed Posture	Corrected Posture	Z	*p*
Right internal oblique	100.00 ± 0.00	106.89 ± 59.79	−0.625	0.247
Left internal oblique	100.00 ± 0.00	116.94 ± 30.45	−2.513	0.058
Right multifidus	100.00 ± 0.00	124.14 ± 70.46	−0.599	0.517
Left multifidus	100.00 ± 0.00	133.06 ± 100.58	−0.651	0.500
Right rhomboid	100.00 ± 0.00	129.91 ± 56.59	−2.058	0.010 *
Left rhomboid	100.00 ± 0.00	112.39 ± 34.63	−2.237	0.046 *
Right serratus anterior	100.00 ± 0.00	83.07 ± 53.02	−1.726	0.130
Left serratus anterior	100.00 ± 0.00	77.88 ± 31.17	−2.518	0.011 *
Right external oblique	100.00 ± 0.00	109.43 ± 43.10	−1.342	0.151
Left external oblique	100.00 ± 0.00	102.84 ± 41.44	−1.138	0.359

Results marked with * are statistically significant for *p* < 0.05.

## Data Availability

The data collected for this study are available from the corresponding author on reasonable request due to the fact that they contain (anonymized) patient results.
